# Insights into trunks of *Pinus cembra* L.: analyses of hydraulics via electrical resistivity tomography

**DOI:** 10.1007/s00468-020-01976-x

**Published:** 2020-04-16

**Authors:** Adriano Losso, Julia Sailer, Andreas Bär, Andrea Ganthaler, Stefan Mayr

**Affiliations:** 1 Department of Botany, University of Innsbruck, Sternwartestraße 15, 6020 Innsbruck, Austria; 2 Hawkesbury Institute for the Environment, Western Sydney University, Richmond, NSW 2753 Australia

**Keywords:** Electrical resistivity tomography, Elevational gradient, *Pinus cembra*, Seasonal changes, Stone pine

## Abstract

**Key message:**

The lack of elevational changes in electrical resistivity in *Pinus cembra* trunks indicated consistent growth and hydraulics across elevations. Though, electrical resistivity tomograms exhibited pronounced temperature-driven seasonal changes.

**Abstract:**

Alpine conifers growing at high elevation are exposed to low temperatures, which may limit xylogenesis and cause pronounced seasonal changes in tree hydraulics. Electrical resistivity (ER) tomography enables minimal invasive monitoring of stems in situ. We used this technique to analyze *Pinus cembra* trunks along a 400 m elevational gradient up to the timberline and over seasons. Furthermore, ER data of earlywood across tree rings were compared with the respective specific hydraulic conductivity (*K*_S_), measured on extracted wood cores. ER tomograms revealed pronounced changes over the year and a strong correlation between average ER (ER_mean_) and air and xylem temperatures. Surprisingly, no elevational changes in ER_mean_, earlywood ER or *K*_S_ were observed. ER data corresponded to variation in earlywood *K*_S_, which decreased from the youngest (ca. 4–5 cm^2^s^−1^ MPa^−1^) to the oldest tree rings (0.63 ± 0.22 cm^2^s^−1^ MPa^−1^). The lack of changes in ER data and earlywood *K*_S_ along the study transect indicated consistent growth patterns and no major changes in structural and functional hydraulic traits across elevation. The constant decrease in earlywood *K*_S_ with tree ring age throughout all elevations highlights the hydraulic relevance of the outermost tree rings in *P. cembra*. Seasonal measurements demonstrated pronounced temperature effects on ER, and we thus recommend a detailed monitoring of trunk temperatures for ER tomography.

**Electronic supplementary material:**

The online version of this article (10.1007/s00468-020-01976-x) contains supplementary material, which is available to authorized users.

## Introduction

Conifers growing in alpine ecosystems face limitations in structural and functional traits due to the decrease in temperature with increasing elevation (Körner 2003). Temperature-related restrictions of wood formation in below- and aboveground biomass (Alvarez-Uria and Körner 2007) also affect xylem hydraulics. For instance, Rossi et al. (2007) and Petit et al. (2011) demonstrated reduced annual increment in basal stem area (see also Körner 2003; Gamache and Payette 2004) as a consequence of limited xylogenesis, which may impair hydraulics of conifers growing at high elevation. Though low temperatures at high elevation do not influence plant–water relations via xylogenesis, decreasing temperatures limits the transport of water by increasing its viscosity (about 2.4% per Kelvin; Tyree and Zimmermann 2002), and when the freezing point is reached, causes the breakdown of water supply by ice formation (Burke et al. 1986; Sakai and Larcher 1987; Mayr and Charra-Vaskou 2007). Moreover, extreme temperatures, high rates of temperature changes, frost drought and freeze–thaw events can lead to a reduction of the overall plant hydraulic efficiency (Groß et al. 1991; Mayr et al. 2003a; Mayr and Sperry 2010; Mayr and Zublasing 2010) and dramatically increase the risk of embolism formation (Mayr et al. 2006). Thus, trees and especially conifers growing at high elevation are interesting objects for hydraulic studies, exhibiting pronounced contrasts between winter and summer water relations (Losso et al. 2017, 2018; Mayr et al. 2020). Several hydraulic studies have been conducted on high elevation trees but were mainly performed on branches (e.g. Mayr et al. 2003b, 2014; Losso et al. 2016), which enable easy sampling and sufficient material for destructive methods. In contrast, insights into functional aspects of trunks of adult trees are (as in studies of other forest systems) scarce.

In the present study, we used the electrical resistivity (ER) tomography, which allows detailed, minimal invasive assessments of tree stems *in natura* (Tattar et al. 1972; Shigo and Shigo 1974; Shortle 1982; Just and Jacobs 1998; Al Hagrey 2007; Bär et al. 2019b). It is based on analysis of the cross-sectional resistivity distribution by generating an electrical field and subsequent measurement of the electrical conductivity (and ER as its reciprocal). The distribution of resistivities is finally visualized in ER tomograms, whereby the spatial resolution depends on the number of measurement points and size of trees (typically in the range of few mm/cm in small/large trunks). Cross-sectional ER patterns are caused by the varying electrical properties of wood, which are mainly influenced by moisture content, electrolyte concentration and wood density (Al Hagrey 2007; Bieker et al. 2010; Bieker and Rust 2010; Guyot et al. 2013). Recently, Bär et al. (2019a, b) reported wood moisture content of conifers to be the main factor influencing ER patterns, which thus accurately display sapwood areas. Ganthaler et al. (2019) showed that average ER values of conifer trunks are correlated with shoot water potential and trunk temperature. While ER distribution patterns do not change above the freezing point, average ER values decrease exponentially from 0 to 30 °C (Ganthaler et al. 2019; see also Luo et al. 2019). Thus, temperature effects should be negligible as long as small changes in temperature occur (e.g. measurements at comparable days and daytime), while seasonal differences in temperature may be relevant for long-term studies. Considering potential temperature effects (e.g. by constant sampling daytime or by calculating a temperature correction of all ER values), ER tomography would then allow insights into the overall water content of the xylem as well as detailed insights into cross-sectional variation in water content. With respect to trees growing at higher elevation (see above), ER tomography should also allow to detect possible elevational changes (related to limitations by, e.g. frost drought, freeze–thaw events, short vegetation period, mechanical loads due to snow and wind; see above) in xylem structure and/or function.

In this study, we selected *Pinus cembra* as a typical alpine conifer growing from 1200 m a.s.l. up to the Central European Alps timberline (ca. 2000–2200 m a.s.l.). The species is well adapted to alpine sites and is considered as comparably drought resistant (Mayr et al. 2006; Losso et al. 2016). We aimed at analyzing the following aspects of trunk water relations based on ER tomography measurements: (1) seasonal changes in ER levels and patterns were studied to test if known pronounced seasonal differences in tree water relations can be monitored by ER tomography; (2) elevational variation in ER tomograms of trunks was analyzed to detect potential elevational changes indicating structural or functional limitation; (3) ER values and xylem hydraulic conductivity (*K*_S_) were correlated across tree rings to link patterns in water content (as indicated by ER tomography) to xylem hydraulic function. We expected ER tomograms to reveal pronounced seasonal and elevational variation in stem water content and cross-sectional patterns to reflect radial profiles in *K*_S_. Changes over seasons and across elevations should thereby reflect the contrasting hydraulic situation during winter and summer, and the limitations in xylem growth and/or hydraulics towards the timberline, respectively. Expected ER–K_S_ relationships might not only help to understand these limitations and/or adaptations, but also be of general interest for sap wood analysis as they would enable an estimation of its functional performance.

## Material and methods

### Plant material

Measurements were performed on trunks (and extracted wood cores) of *P. cembra* L. trees growing near Praxmar (47° 09′ N/11° 07′ E), on a southeast exposed slope in the Tyrolean Central Alps (Austria). Vital trees with no visible damages, straight stems, and preferably circular trunks were selected for measurements.

### Seasonal course

Four young *P. cembra* specimens of similar size and habit (height ca. 2.5 m and diameter at breast height ca. 5–6 cm) growing at 1700 m a.s.l. were selected. In September 2012, nail probes were installed around the trunk circumference at 24 measuring points (MP), 40 cm above the ground (Al Hagrey 2007; Bär et al. 2019a; Ganthaler et al. 2019). Nails (length 5 cm) were installed around the trunk circumference with equal distance to each other (counterclockwise numbering with north orientation of MP 1), until contact to the sapwood was established. Due to the small and homogeneously round shape of stems, a circular cross-section was assumed. From September 2012 to October 2013, at regular time intervals, electrical resistivity (ER) measurements were performed on each tree. The same set of nails was used over winter and new sets were installed during the vegetation period in July and August 2013. The relatively small size of trees under study did not allow repeated electrode substitution during winter and spring. During wintertime, relatively warm days were selected for measurements, which were always carried out around 1 pm. All nails were connected via electrodes to a 24-channel resistivity system (PiCUS: TreeTronic, Argus Electronic Gmbh, Rostock, Germany) and electrical voltages (voltage levels between 2 and 4) were applied systematically to all MP (overall duration of measurements was less than 5 min). Data of the electrical field were sent to the software on a laptop, with which the cross-sectional distribution of ER was calculated, and the respective tomogram generated. Spatial distribution of resistivities was reconstructed using an inversion scheme based on a finite element simulation operating with regularly arranged tetrahedrons (Günther 2004; Günther et al. 2006; Rücker et al. 2006). Tomograms were constructed by the software based on triangle areas, where each triangle was colored according to its resistivity for a better visualization of patterns. Information on triangle size, position and ER value was calculated from overlapping resistivity values at each point by the software, and respective data were exported. Triangle areas varied depending on their radial position, and the weighted electrical resistivity (ER_w_; Ωm) was thus calculated for each triangle as

1 $${\text{ER}}_{{\text{w}}} \, = \,\left( {{\text{ER}}\, \times \,A} \right){/}A_{{{\text{mean}}}}$$

where *A* (cm^2^) and *A*_mean_ (cm^2^) are the individual triangle area and the mean area of all triangles, respectively. The average ER of each entire cross-section (ER_mean_) was calculated as the mean of ER_w_ of all triangles. ER profiles (oriented from south to north) were created for each tomogram by excerpting ER values along sectors (width = 5% of stem diameter) spanning the tomogram.

From September 2012 to October 2013, air temperature data were recorded with a weather station (temperature and humidity sensor EMS33, precipitation sensor MetOne 370/376, and datalogger ModuLog 3029 from Environmental Measuring Systems, Brno, Czech Republic) located at about 10 m distance to trees under study. For xylem temperature data, type T thermocouples connected to the datalogger were inserted in the stem at 30 cm above the ground. Measurements were taken at 1-min intervals and 15-min mean values were stored. Average values (1 h) were calculated for the time at which ER measurements were performed.

### Elevational transect

In October 2013, 11 *P. cembra* trees with similar habit (height ca. 3 m and diameter at breast height ca. 7–8 cm) were selected along a south-east exposed 400-m elevational transect spreading from 1674 to 2081 m a.s.l. For each tree, ER measurements were performed on the trunk at 60 cm above ground as described above (nails were removed after measurements). Maximum (ER_max_) and minimum (ER_min_) ER_w_ values of included triangles were excerpted from the same ER profiles (from south to north) created for each tomogram by excerpting ER values along sectors (width = 5% of stem diameter) spanning the tomogram. The exact position and elevation of each sampled tree individual were recorded with a GPS tool (GPSMAP 64st, Garmin GmbH, Garching, Germany).

### Radial patterns

For six specimens growing at the highest (i.e. 2081, 2002 and 1920 m a.s.l) and lowest elevation (i.e. 1689, 1686 and 1674 m a.s.l.), extracted ER profiles were related to specific hydraulic conductivity (*K*_s_) measurements.

**Fig. 1:**
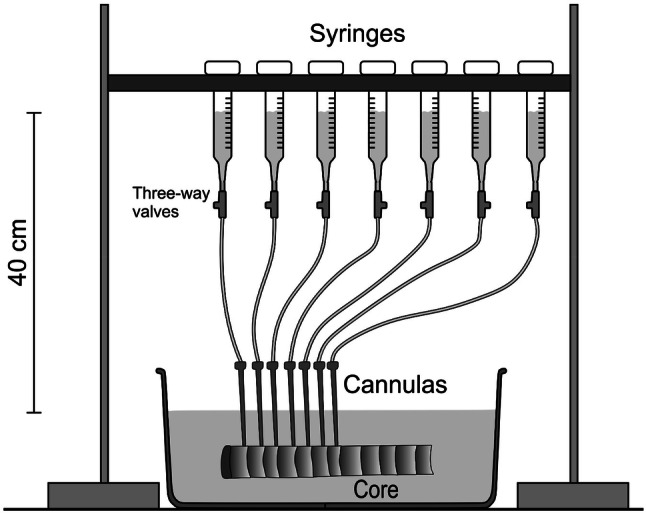
Conductivity measurements on wood cores. A set of 1-mL syringes was mounted 40 cm above the water level of a basin with prepared radial trunk cores (diameter 1 cm). Syringes were connected to sharpened, water-filled steel cannulas, which were inserted in the earlywood of consecutive tree rings. Three-way valves enabled to fill silicone tubes and adjust the height of water columns

Therefore, from the south side of the trunk of the same specimens, a core (diameter: 1 cm; length: 6.07 ± 0.32 cm) was collected at the same height where ER measurements were performed with an increment borer (Haglöf, Långsele, Sweden). It was immediately packed in black plastic bags, and transported to the laboratory, where cores were stored in a freezer at − 19 °C prior measurements. For specific hydraulic conductivity (*K*_s_) measurements, cores were thawed immersed under water and, while constantly wetting them with a damp brush, transversely smoothed with a sliding microtome (Sledge Microtome G.S.L. 1, Schenkung Dapples, Zürich, Switzerland) to obtain a plane cross-sectional area at the upper and lower surface (with respect to the axial direction; Fig. 1). Samples were then individually placed in a beaker filled with distilled, filtered (pore size 0.22 µm), and degassed water containing 0.005% (v/v) ‘Micropur’ (Katadyn Products, Wallisellen, Switzerland to prevent microbial growth) and vacuum infiltrated to remove embolism potentially induced during sample preparation.

*K*_s_ of the earlywood of individual tree rings was measured with a modified ‘micro-Sperry apparatus’ (Mayr and Cochard 2003; see also Fig. 1), which allows conductivity measurements on very small sample areas. In brief, sharpened, water-filled steel cannulas (1.1 × 19; ECOFLO^®^Dispomed, Witt oHG, Germany) were inserted for 2–4 mm in the earlywood of the last 12 years, respectively, next to the tree ring boundary. Each cannula was connected to a 1-mL syringe (Omnifix^®^-F Solo, Braun Melsungen AG, Germany) over a small water-filled silicone tube and a three-way valve, which allowed the syringe water column to be set to a defined starting point before measurements. Flow was calculated from volume changes within the syringe capillaries during the measurement time (10 min). Measurement pressure (i.e. height of water column) was set to about 4 kPa and the same water solution as for vacuum infiltration was used (see above). Eight measurements could be performed in parallel, before cannulas were placed in the consecutive tree rings. The cross-sectional area inside the cannula (= conductive wood surface) was 0.95 mm^2^. *K*_S_ was thus calculated as

2 $$K_{{\text{S}}} \, = \,Q\, \times \,{\text{l}}/\left( {A_{{\text{c}}} \, \times \,\Delta P} \right),$$

where *Q* is the volume flow rate (m^3^ s^−1^), l the length of the segment (m), *A*_c_ the cross-sectional area situated inside the cannula (m^2^), and Δ*P* the mean pressure head (MPa) calculated from the height of the water column at start and end of flow measurements. Calculations were corrected to 20 °C to account for changes in fluid viscosity with temperature. *K*_S_ values of trees growing at the highest and lowest elevation were averaged per tree ring. Similarly, for each tree, the means of ER values of triangles, whose centers were located at ± 5 mm in tangential direction from the injection point along the south–north sector, were calculated and used for correlation analysis between *K*_S_ values and ER within the earlywood.

### Statistical analysis

Correlation analysis was based on Pearson product-moment correlation. All tests were conducted using SPSS software v. 21.0 (SPSS Inc., Chicago, IL) at a probability level of 5%.

## Results

### Seasonal course

**Fig. 2:**
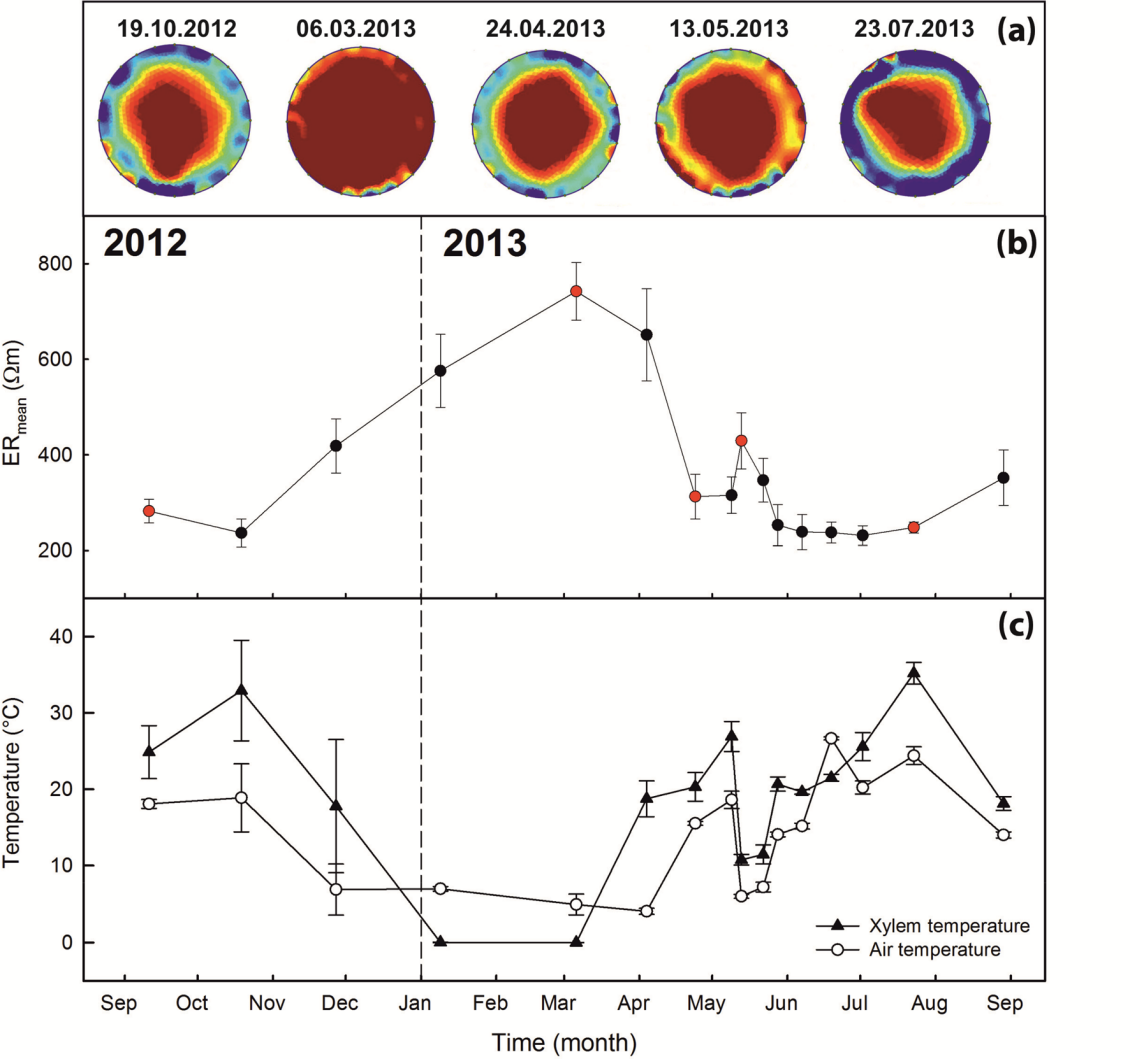
Annual course of cross-sectional electrical resistivity (ER). **a** ER tomograms of the trunk of a representative *P. cembra* tree measured at five different dates in Praxmar, Austria (1700 m a.s.l.). Areas of high ER are indicated by red color while areas of low resistivity are indicated by blue color. Note that the limit of the displayed resistivity ranges was set manually (80–400 Ωm) to optimize visualization, and ER_min_ and ER_max_ values may exceed these limits. **b** Mean electrical resistivity (ER_mean_) measured from September 2012 to September 2013 on four *P. cembra* trees. Red dots indicate measurement dates of tomograms in **a**. **c** Air (open circles) and xylem temperatures (close triangles) during ER measurements [average values (1 h) were calculated for the time at which ER measurements were performed]. Mean ± SE

Stem tomograms and respective ER_mean_ (Fig. 2) changed remarkably across seasons, while radial patterns remained rather constant (Supplementary Material 1). Highest resistivity values were observed in winter months (i.e. from December 2012 to April 2013). ER_mean_ progressively increased from autumn (October: 236.5 ± 29.1 Ωm), reached its maximum at the end of winter (March: 742.3 ± 60.7 Ωm) and rapidly decreased at the end of April. In May 2013, ER_mean_ increased again (429.3 ± 58.9 Ωm), before it finally decreased again and remained constantly low over summer months (from May to August at ca. 250–350 Ωm). At the end of summer/beginning of autumn 2013, values started to raise again. ER_mean_ was negatively correlated to seasonal courses in air and xylem temperatures (*P* < 0.001, *r* = − 0.788 and *P* = 0.001, r = − 0.755, respectively; Fig. 2b, c).

### Elevational transect

**Fig. 3:**
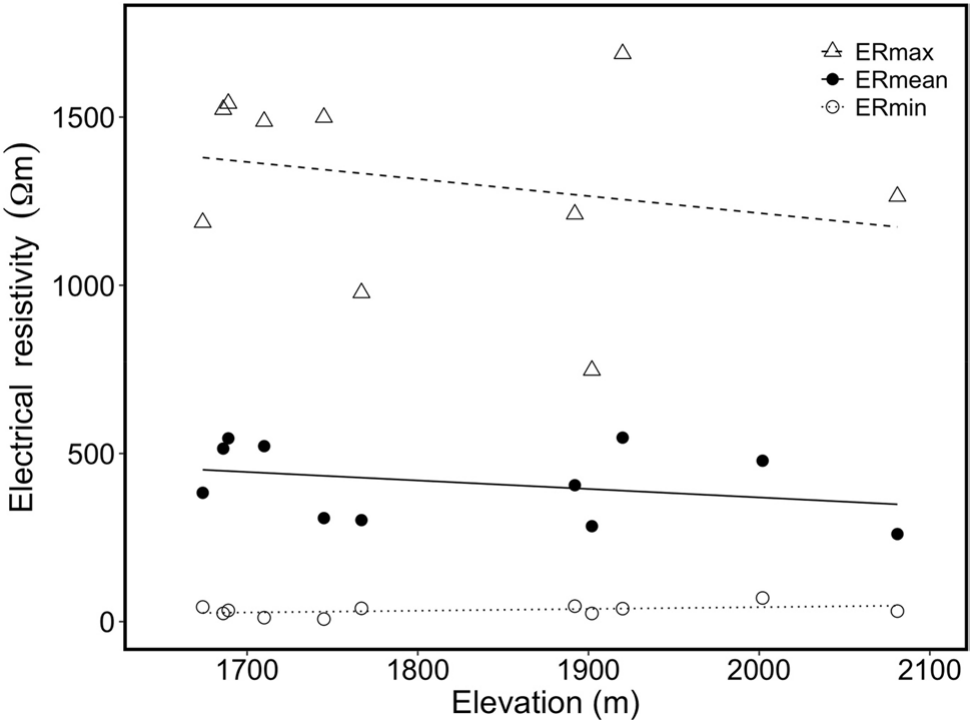
Elevational changes in cross-sectional electrical resistivity. Minimum (ER_min_; open circles), maximum (ER_max_; open triangles) and mean (ER_mean_; full circles) electrical resistivity of trunks of *P. cembra* growing at different elevations. Lines indicate linear regressions of ER_min_ (*P* = 0.191, *R*^2^ = 0.186), ER_max_ (*P* = 0.282, *R*^2^ = − 0.056) and ER_mean_ (*P* = 0.339, *R*^2^ = 0.100)

**Fig. 4:**
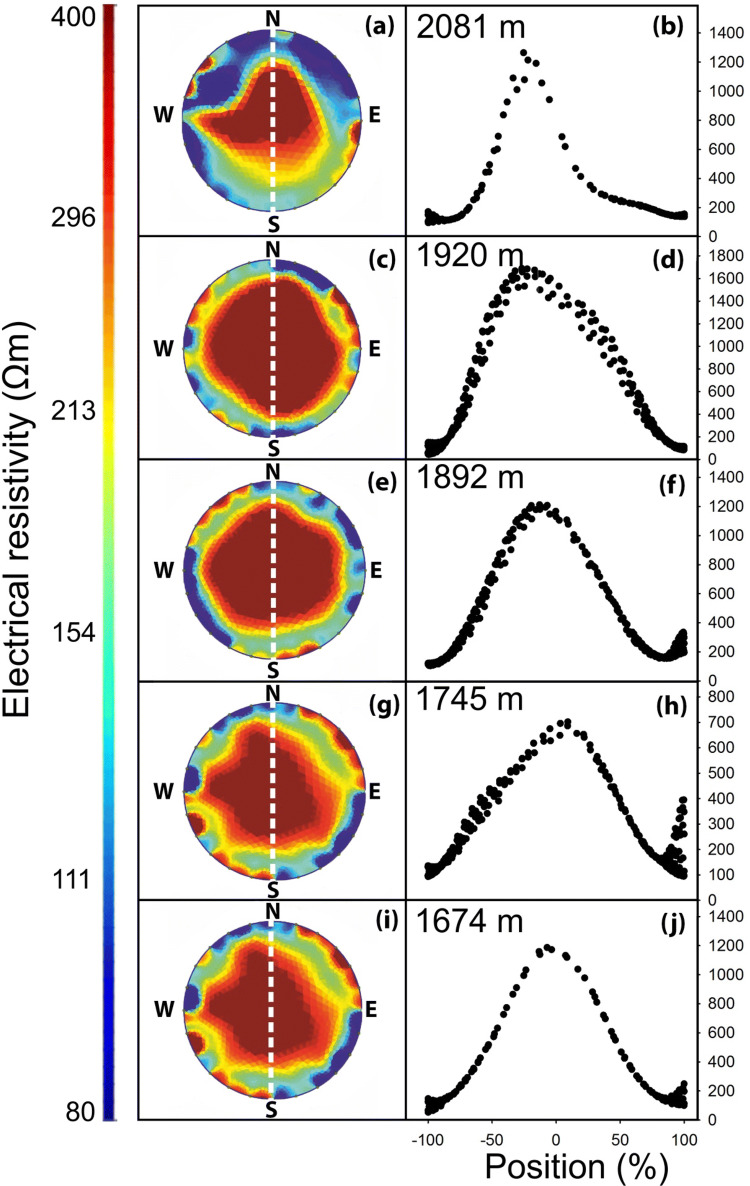
Electrical resistivity (ER) tomography at different elevations. This figure shows five representative trees (out of 11 analyzed trees) growing at 2081 (**a**, **b**), 1920 (**c**, **d**), 1892 (**e**, **f**), 1725 (**g**, **h**) and 1674 m a.s.l. (**i**, **j**), from which ER tomograms and profiles of trunks of *P. cembra* trees. Areas of high resistivity in tomograms are indicated by red color while areas of low resistivity are indicated by blue color. Note that the limit of the displayed resistivity ranges was set manually to optimize visualization, and ER_min_ and ER_max_ values may exceed these limits. For each tomogram, ER values were excerpted along a chosen profile (dashed white line, width 1 cm). Absolute ER values in profiles are displayed according to their relative position (0% = trunk center, 100% = periphery of youngest tree ring)

Minimum (ER_min_; *P* = 0.191, *r* = 0.426), maximum (ER_max_; *P* = 0.282, *r* = − 0.357) and mean (ER_mean_; *P* = 0.319, *r* = − 0.319) electrical resistivity values were not correlated with elevation (Fig. 3). ER_mean_ and ER_max_ tended to decrease with increasing elevation, while variation in ER_min_ was small. As visible from representative trees in Fig. 4, patterns in tomograms of studied specimens were similar and did not indicate any elevational change. At highest elevation, trees already showed some cripple growth (i.e. bending of upper stem sections non-uniform of crown), which was also visible in non-symmetric tomogram patterns (Fig. 4). However, in all tomograms, a clear differentiation of sap- and heartwood was visible.

### Radial patterns

**Fig. 5:**
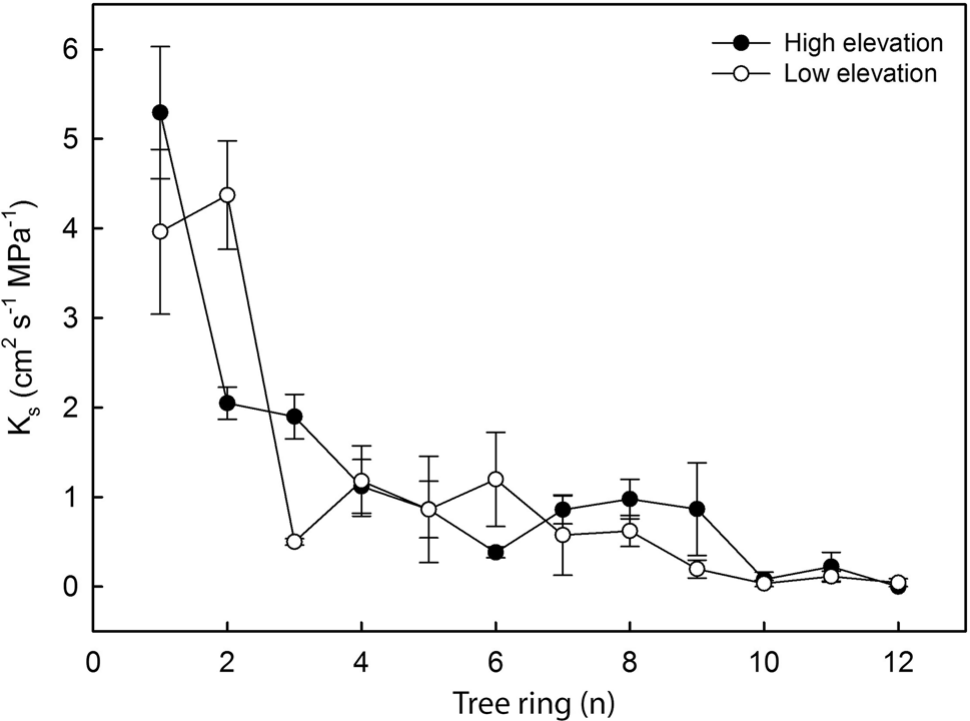
Hydraulic conductivity across tree rings. Specific hydraulic conductivity (*K*_S_) of the 12 youngest tree rings measured on a core extracted from the trunk of *P. cembra* trees growing at high (ca. 2000 m a.s.l.; full circles) and low elevations (ca. 1600 m a.s.l.; open circles). Mean ± SE

**Fig. 6:**
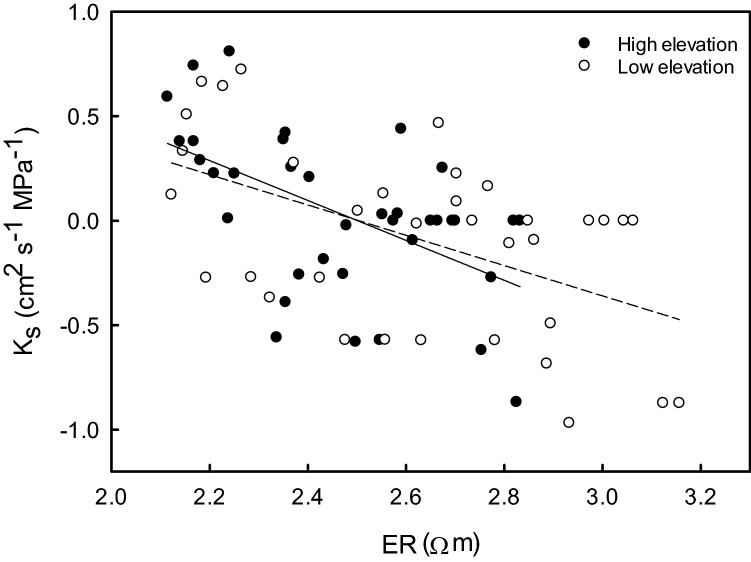
Hydraulic conductivity of tree rings *versus* electrical resistivity. Log–log plot of specific hydraulic conductivity raw data (*K*_S_) *versus* mean electrical resistivity (ER) of trees growing at high (ca. 2000 m a.s.l.; full circles) and low elevation (ca. 1600 m a.s.l.; open circles). Solid and dashed lines indicate linear regressions for high (*P* = 0.001, *R*^2^ = 0.288) and low elevation trees (*P* = 0.002, *R*^2^ = 0.261), respectively

In all analyzed trees, earlywood *K*_S_ decreased from youngest to oldest tree rings (Fig. 5). The youngest tree rings showed much higher *K*_S_ (on average 3.92 ± 0.61 cm^2^ s^−1^ MPa^−1^) than older rings. At low elevation, also the second tree ring exhibited high *K*_s_, while it was less than half of the outermost tree ring at high elevation. *K*_S_ of tree rings older than 4 years was on average 0.63 ± 0.22 cm^2^ s^−1^ MPa^−1^. In tree rings older than 10 years, no flow could be detected in high elevation trees and only very low K_S_ (0.05 ± 0.05 cm^2^ s^−1^ MPa^−1^) was found at low elevation. As demonstrated in Fig. 6, *K*_S_ was negatively correlated with ER in both high (*P* = 0.001, r = -− 0.537) and low elevation trees (*P* = 0.002, *r* = − 0.511). Overall, *K*_S_, ER_mean_ and their interrelation were similar at low and high elevation.

## Discussion

ER tomography enabled in situ insights into cross-sectional patterns of *P. cembra* trunks. Tomograms exhibited pronounced seasonal changes, which, as explained below, were predominantly related to temperature effects but not to structural or functional changes over the year. Remarkably (and in contrast to our hypotheses), we did not find elevational changes in tomogram patterns or ER. Also, trees showed a similar radial profile in ER, which corresponded well to *K*_s_, despite contrasting climatic and thus growth conditions at low and high elevation.

Previous studies demonstrated that species of the Pinaceae family (Bieker and Rust 2010; Guyot et al. 2013; Bär et al. 2019a) usually exhibit a very clear ER tomography pattern with distinct heartwood and sapwood areas separated by a comparably small transition zone. Sapwood typically has higher moisture and electrolyte content (Just and Jacobs 1998; Guyot et al. 2013), and therefore, lower ER_mean_ than the heartwood. This pattern was also observed in *P. cembra* during the vegetation period (Supplementary Material 1), though absolute ER values changed dramatically over seasons. While ER_mean_ was around 300 Ωm in the vegetation period, it increased to more than 700 Ωm during winter. It is obvious from Fig. 2 (and Supplementary Material 1) that this was based on a strong temperature effect, with highest ER_mean_ probably caused by frozen trunks, as in ice the mobility of electrons is strongly reduced. In spring 2013, a cold period (May) caused another increase in ER_mean_, and accordingly ER_mean_ and air/xylem temperature were negatively correlated (see “Results”). In laboratory tests, Ganthaler et al. (2019) demonstrated that temperature directly affects the measured ER and reported for six species, including *P. cembra*, an about 2.5-fold increase in ER_mean_ between 30 and 0 °C. In the present study, this increase was even higher, and we suppose that this indicates two additional processes: First, conifers at higher elevation suffer from frost drought during winter, which induces low water potentials and respective low water contents (e.g. Tranquillini 1957; Groß et al. 1991; Mayr et al. 2003b, 2006), which may further increase ER_mean_. According to our hypothesis, seasonal changes in tree water status thus influenced ER tomograms, but effects were masked by the pronounced temperature effect. Second, the repeated use of nail probes installed in September 2012 might have affected ER_mean_. Ganthaler et al. (2019) pointed out that resin production and wound reactions may reduce the electric connection to trunk tissues over time and, in consequence, affect tomography measurements. More ER tomograms of conifer species with high resin content, such as *P. cembra*, are required to estimate the relevance of wound reactions. In each case, the present study underlines the importance of considering potential temperature effects (and/or artifacts) for the interpretation of ER tomograms, and we also encourage further studies investigating the influence of ice patterns within stems of larger trees. Notably, temperature effects were not relevant in our analysis on potential elevational variation as measurements were performed at similar temperatures within few, sunny days in October 2013.

Along the entire transect under study, *P. cembra* specimens exhibited neither relevant changes in ER_mean_ (Fig. 3) nor in tomogram patterns (Fig. 4). Again, all trees exhibited cross-sectional ER distribution typical for vital conifers (Bieker and Rust 2010; Guyot et al. 2013). Only trees at the highest elevation occasionally showed asymmetric patterns (see Fig. 4a), which might be related to characteristic cripple growth due to mechanical stress at the timberline. However, the overall similar tomograms indicate a lack of elevational changes in major structural or functional traits of *P. cembra* trunks. This is surprising as growth conditions and xylogenesis are known to be limited toward the timberline (see “Introduction”). Though, *P. cembra* is a species well adapted to the harsh environmental conditions at high elevation, and as reflected by tomograms, limitations in growth patterns or structural and functional hydraulic traits along the study transect might be small. It remains to be studied if other species (see, e.g. Mayr et al. 2010; Charra-Vaskou et al. 2012) or transects of wider elevational range exhibit changes in ER tomograms, indicating limitation and/or adjustments.

We also observed similar *K*_S_*versus* ER relations (Fig. 6) at low and high elevation: ER tomography indicated highest water contents in trunk periphery, which is expected to contribute most to both water storage and sap flow in *P. cembra*. The latter was supported by *K*_s_ measurements in the earlywood (which conducts most of the water within a tree ring) of tree rings of extracted wood cores (Fig. 5). *K*_s_ was highest in the youngest tree ring, and also the second was highly conductive. The remaining ten tree rings showed ca. 15% of the conductivity of the first tree ring. It is known that water transport in mature conifers is normally limited to the youngest 10–20 tree rings and reaches the highest flow rates in the recently formed sapwood rings (Cermak et al. 1992). The functional dominance of the two outermost tree rings in all trees indicates that the sapwood–heartwood transformation in *P. cembra* follows a fixed program, which is not influenced by climate or growth conditions. Though, for trees growing at high elevation, the biggest decrease in *K*_s_ occurred between the first and the second tree rings, while, at low elevation, it occurred between the second and the third tree rings (Fig. 5). At high elevation, when cripple growth causes smaller crowns, this may lead to increased Huber values and leaf-specific conductivities (Tyree and Ewers 1991; Tyree and Zimmermann 2002; Mayr et al. 2003c) and thus be advantageous for trees.

The present study demonstrates that ER tomography is a useful tool for analyzing structural and functional traits of tree trunks, but temperature effects may substantially limit this method. Unless relevant temperature variations can be excluded, measurements of xylem temperatures and corrections of ER calculations are thus strongly recommended (Ganthaler et al. 2019; Luo et al. 2019). To account for complex temperature patterns, e.g. due to intense sap flow, sun exposure or winter freezing dynamics, even detailed temperature profiles across the xylem cross-section will be necessary. ER tomography then might even allow estimations of sapwood conductivity as demonstrated by *K*_S_*versus* ER correlations and thus contribute important information to sap flow measurements. In the case of *P. cembra*, this technique revealed only small variability in ER_mean_ and within-trunk ER patterns across elevation indicating limited variation in xylem structure and hydraulics. Further studies are required to test the species specificity of observed elevational patterns and of correlations between hydraulic traits and ER tomography measurements.

### Author contribution statement

SM planned and designed the present study. JS performed data collection. AL, AB, AG, JS and SM performed data analyses and interpretation. The manuscript was prepared by AL with contributions from all other authors.

## Electronic supplementary material

Below is the link to the electronic supplementary material.

Supplementary file1 (JPG 2751 kb) Electrical resistivity (ER) tomography over seasons. ER tomograms and profiles of the trunk of a representative P. cembra tree measured in October 2012 (a), March (b), June (c) and July 2013 (d) are given. Areas of high resistivity in tomograms are indicated by red colors while areas of low resistivity are indicated by blue colors. Note that the limit of the displayed resistivity range was set manually to optimize visualization, and ERmin and ERmax values may exceed these limits. For each tomogram, ER values were excerpted along a chosen profile (dashed white line, width 5% of stem diameter). Absolute ER values in profiles are displayed according to their position ((0 cm = trunk center)
