# Supplementary figures and images for: Insights into trunks of Pinus cembra L.: analyses of hydraulics via electrical resistivity tomography
Source: Trees (Berl West). 2020 Apr 16;34(4):999–1008. doi: 10.1007/s00468-020-01976-x (PMC7437670; doi:10.1007/s00468-020-01976-x)

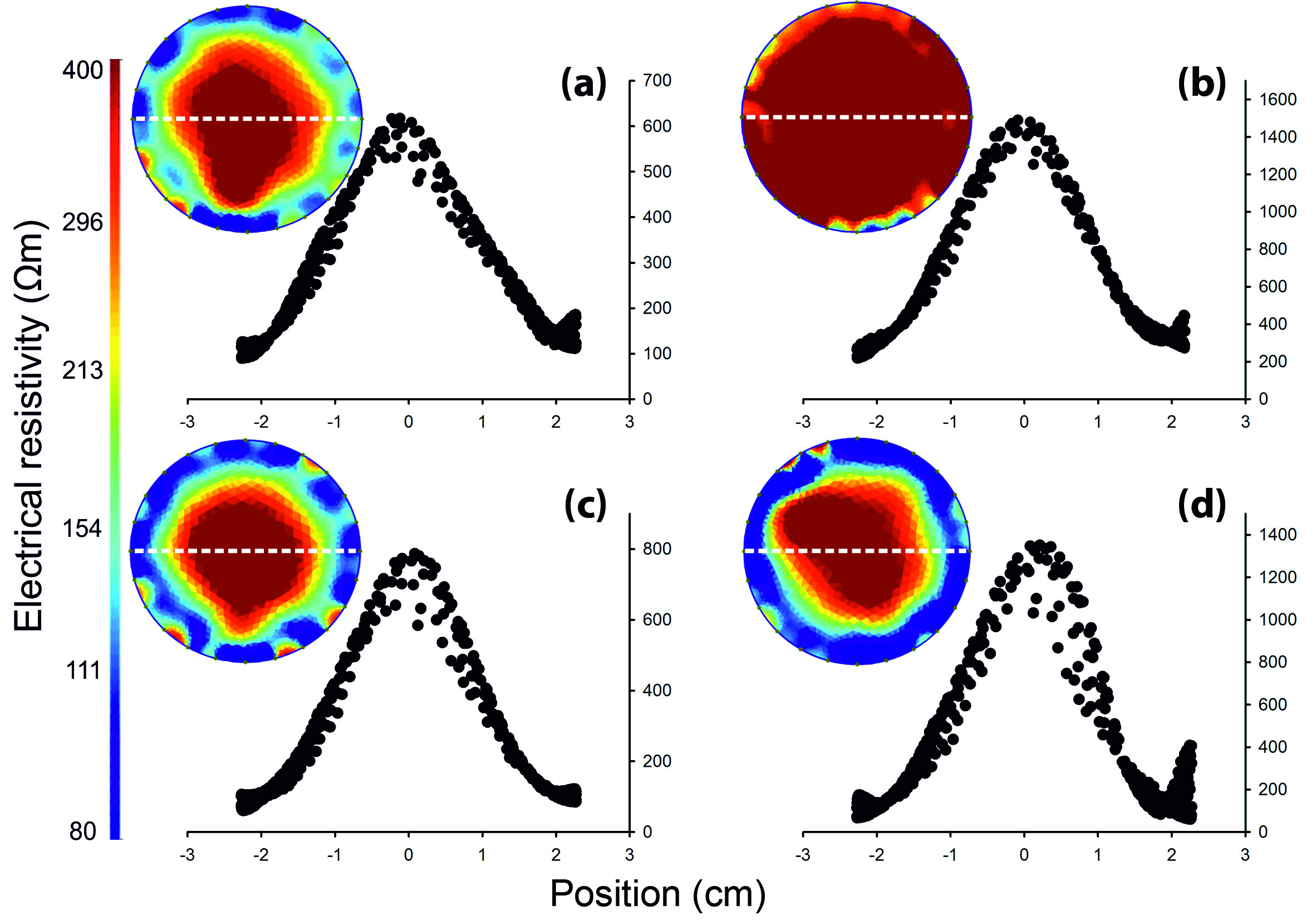

Supplement: Supplementary file 1 — Supplementary file1 (JPG 2751 kb) Electrical resistivity (ER) tomography over seasons. ER tomograms and profiles of the trunk of a representative P. cembra tree measured in October 2012 (a), March (b), June (c) and July 2013 (d) are given. Areas of high resistivity in tomograms are indicated by red colors while areas of low resistivity are indicated by blue colors. Note that the limit of the displayed resistivity range was set manually to optimize visualization, and ERmin and ERmax values may exceed these limits. For each tomogram, ER values were excerpted along a chosen profile (dashed white line, width 5% of stem diameter). Absolute ER values in profiles are displayed according to their position ((0 cm = trunk center) [file 468_2020_1976_MOESM1_ESM.jpg]
